# Eating Competence Is Associated with Lower Prevalence of Obesity and Better Insulin Sensitivity in Finnish Adults with Increased Risk for Type 2 Diabetes: The StopDia Study

**DOI:** 10.3390/nu12010104

**Published:** 2019-12-30

**Authors:** Tanja Tilles-Tirkkonen, Kirsikka Aittola, Reija Männikkö, Pilvikki Absetz, Marjukka Kolehmainen, Ursula Schwab, Jaana Lindström, Timo Lakka, Jussi Pihlajamäki, Leila Karhunen

**Affiliations:** 1 Department of Clinical Nutrition, Institute of Public Health and Clinical Nutrition, University of Eastern Finland, 70211 Kuopio, Finland; kirsikka.aittola@uef.fi (K.A.); reija.mannikko@uef.fi (R.M.); pilvikki.absetz@gmail.com (P.A.); marjukka.kolehmainen@uef.fi (M.K.); ursula.schwab@uef.fi (U.S.); jussi.pihlajamaki@uef.fi (J.P.); leila.karhunen@uef.fi (L.K.); 2 Collaborative Care Systems Finland, 00270 Helsinki, Finland; 3 Department of Medicine, Endocrinology and Clinical Nutrition, Kuopio University Hospital, 70029 KYS Kuopio, Finland; 4 Department of Public Health Solutions, National Institute for Health and Welfare, 00271 Helsinki, Finland; jaana.lindstrom@thl.fi; 5 Institute of Biomedicine, School of Medicine, University of Eastern Finland, 70211 Kuopio, Finland; timo.lakka@uef.fi; 6 Department of Clinical Physiology and Nuclear Medicine, Kuopio University Hospital, 70029 KYS, Kuopio, Finland; 7 Kuopio Research Institute of Exercise Medicine, 70100 Kuopio, Finland

**Keywords:** diet, eating behaviour, eating competence, glucose metabolism, insulin metabolism, lifestyle, metabolic syndrome, type 2 diabetes

## Abstract

A healthy diet prevents type 2 diabetes but is often difficult to adhere to. This cross-sectional study aimed to investigate whether eating competence is associated with diet or risk factors and prevalence of type 2 diabetes in individuals screened for type 2 diabetes risk. Eating competence is an indicator of food acceptance, positive attitudes, internal regulation and contextual skills related to food and eating. In total, 3147 Finnish adults aged 18–74 at an increased risk for type 2 diabetes identified via online risk screening participated in the baseline examinations of the Stop Diabetes (StopDia) study. The participants filled out the digital questionnaire on food intake, physical activity and sleep, and the Satter Eating Competence Inventory 2.0^TM^ (ecSI 2.0^TM^). In addition, anthropometric and laboratory measurements were performed at primary healthcare centres. Eating competent individuals (37%, classified by ecSI 2.0^TM^) had a better quality of diet (*p* < 0.05 for all). Additionally, eating competence was associated with a lower prevalence of previously undiagnosed type 2 diabetes, abdominal obesity, metabolic syndrome and hypertriglyceridaemia, and with better insulin sensitivity (*p* < 0.05 for all). However, these associations, except for metabolic syndrome, were at least partly mediated by body mass index. Eating competence is associated with a healthy diet and could, thus, in the long term, support the prevention of type 2 diabetes.

## 1. Introduction

Type 2 diabetes can be prevented with lifestyle modification in different populations [1,2,3]. However, the efficacy of lifestyle interventions depends strongly on whether the lifestyle changes are adopted [4]. Finding new, efficient ways to adopt and maintain healthy lifestyles requires improving our understanding of factors associated with health-related behaviours.

One promising approach that has been related to a health-promoting lifestyle is eating competence, which emphasises positive and flexible attitudes towards food and eating [5]. Satter [5] has conceptualized eating competence as consisting of four subcomponents: eating attitudes (e.g., feeling relaxed and comfortable around eating), contextual skills (e.g., planning and having regular meals), food acceptance (e.g., eating a variety of foods) and internal regulation (e.g., eating to satisfaction). Eating competence can be assessed by a validated questionnaire, the Satter Eating Competence Inventory 2.0^TM^ (ecSI 2.0^TM^) [5,6,7,8]. The ecSI 2.0^TM^ is used to classify individuals into eating competent and not eating competent individuals [8].

The Satter Eating Competence Inventory 2.0^TM^ does not specifically emphasise the quality of diet. Still, eating competence has been associated with a better quality of diet [6,7,9,10,11,12]. Eating competent individuals have been found to consume fruit [6,7,10] and vegetables more often [6,7], and have lower sugar intake [9] and greater adherence to a Mediterranean-type diet [10] compared with individuals defined as not eating competent. Eating competence has also been associated with higher physical activity [6,7,13,14] and better sleep quality [13]. Moreover, eating competent individuals have been observed to have a lower body mass index (BMI) [6,7,12,13] and waist circumference [15], lower fasting serum glucose [10], higher plasma HDL (high-density lipoprotein) cholesterol concentrations [10] and lower blood pressure in response to stress [9] compared with not eating competent individuals.

To date, the knowledge about the association of eating competence with the risk of type 2 diabetes is limited [9,10] and the association with glucose tolerance and insulin resistance is unknown. Therefore, we investigated whether eating competence is associated with lifestyle and metabolic risk factors for type 2 diabetes as well as the prevalence of type 2 diabetes in Finnish adults with an increased risk for type 2 diabetes.

## 2. Materials and Methods

### 2.1. Study Design

The present study was based on cross-sectional analyses of the baseline measurements of the Stop Diabetes (StopDia) study that was a one-year RCT (ClinicalTrials.gov registration no. NCT03156478) for individuals at risk for type 2 diabetes [16]. The study consisted of the lifestyle intervention delivered through the digital application and the face-to-face group counselling. The group counselling was performed in local healthcare centres and included six face-to-face sessions. The study was conducted in three regions in Finland: Northern Savo in the east, Southern Carelia in the southeast and Päijät-Häme in the south.

The StopDia study was approved by the Research Ethics Committee of the Hospital District of Northern Savo (statement no: 467/2016). All participants provided their written informed consent. The study was conducted according to the Responsible Conduct of Research guidelines by the Finnish Advisory Board on Research Integrity, and the Declaration of Helsinki.

### 2.2. Study Procedure and Participants

The detailed protocol of the StopDia Study has been published [16]. In brief, the participants were recruited using the digital Finnish Diabetes Risk Score (FINDRISC, see Supplementary Materials) for determining the person’s risk of developing type 2 diabetes within the following 10 years [17]. Several communication channels were used for recruitment that was implemented between March 2017 and February 2018 [16]. The inclusion criteria for the StopDia RCT were (1) age of 18 to 70 years; (2) 12 points or more in the FINDRISC or previous gestational diabetes or repeated impaired fasting glucose (IFG, fasting plasma glucose 6.1–6.9 mmol/L) or impaired glucose tolerance (IGT, 2 h glucose 7.8–11.0 mmol/L in 2 h OGTT (oral glucose tolerance test)); (3) residence in one of the study regions; (4) possibility to use a computer, smartphone or tablet with an Internet connection; (5) having a personal cell phone number; and (6) adequate Finnish language skills. The exclusion criteria were (1) previously diagnosed type 1 or 2 diabetes; (2) pregnancy or breastfeeding; (3) cancer or less than six months since concluding cancer treatment [16].

Eligible individuals were invited to participate in the RCT and were given instructions on how to book an appointment with a study nurse at their local primary healthcare centre [16]. At the first study visit, the participants signed a written informed consent form, and the nurse performed clinical measurements and referred the participants to laboratory measurements. After completing the first study visit, the participants received a personal link by email to the StopDia Digital Questionnaire and were instructed to complete the questionnaire within two weeks.

In total, 3271 individuals enrolled in the baseline clinical measurements; 3134 of them enrolled in the laboratory measurements. The participants of the present cross-sectional study include 3147 individuals who had answered the ecSI 2.0^TM^ within the StopDia Digital Questionnaire, including also participants who were diagnosed with new type 2 diabetes based on the baseline laboratory measurements (*n* = 176) or were over 70 years old (*n* = 7), although they were not randomized to the RCT.

### 2.3. Measurements

#### 2.3.1. Assessments Based on the StopDia Digital Questionnaire: Eating Competence

Eating Competence was measured with the ecSI 2.0^TM^ [5,6,7,8,18], which was translated into Finnish, and a back-translated version was approved by the original authors. The ecSI 2.0^TM^ comprises 16 Likert-scaled items, summed up to yield a total score and four subscale scores [8]. The response options of the questionnaire were always, often, sometimes, rarely and never, and scored 3, 2, 1, 0 and 0 points, respectively [8]. The possible range was 0–48 for the total eating competence score, 0–18 for eating attitudes, 0–15 for contextual skills, 0–9 for food acceptance and 0–6 for internal regulation, as recently suggested by Godleski et al. [19]. Participants with a total eating competence score of 32 or higher were classified as eating competent participant (ECP), and those scoring less than 32 as not eating competent participant (NECP) [6,8]. Cronbach’s alpha coefficients were used to assess internal consistency. Cronbach’s alpha coefficient was 0.83 for the total ecSI 2.0^TM^, 0.77 for eating attitudes, 0.76 for contextual skills, 0.62 for food acceptance and 0.73 for internal regulation.

#### 2.3.2. Sociodemographic Factors, Medical History and the FINDRISC

In the StopDia Digital Questionnaire, information was collected about the respondents’ date of birth, sex, marital status, annual household gross income, education and native country. The questionnaire also included questions on history of diseases diagnosed by a physician, family history of type 2 diabetes, prescribed medications during the previous 12 months, and questions needed to calculate the FINDRISC (daily physical activity, the daily consumption of vegetables, fruit, and berries, history of regular use of antihypertensive drugs, history of elevated blood glucose, family history of diabetes), which had theoretical score range of 0–26 [17].

#### 2.3.3. Dietary Patterns

Food, drink and alcohol consumption was measured with an 18-item food intake questionnaire, which was slightly modified from a previously validated Finnish food intake questionnaire [20]. Dietary factors relevant to the risk of type 2 diabetes were chosen for the analysis from the questionnaire. The continuous variables included the number of servings of vegetarian dishes per week, processed meat products (g/day, all sorts were combined and multiplied by usual serving size), sugary beverages (dL/day, including sugary soft and energy drinks and sugar-sweetened juice), coffee (dL/day) and alcohol intake. The daily intake of alcohol in grams was derived from the alcohol content of the beverages and serving sizes. The categorical variables were categorized into two classes to express more or less healthy dietary patterns utilising the principles of the Finnish nutrition recommendations [21]. The categorical variables were the frequency of having breakfast, lunch and dinner (every weekday or less frequently), eating fast food (<1 portion or ≥1 portions per week), consumption of vegetables, roots, fruit and berries (≥4 portions or <4 portions per day), regular use of oil-based fats (in cooking, as salad dressing, and margarine as a table spread), consumption of nuts and seeds (≥4 portions or <4 portions per week) and sweet desserts (including sweet pastries, pudding and ice cream, <4 portions or ≥4 portions per week).

#### 2.3.4. Sleeping, Smoking and Physical Activity

Sleep duration, defined by factors including usual bedtime and wake-up time on weekdays, and sleep quality ranging from 0 (worst) to 10 (best), was assessed by the Basic Nordic Sleep Questionnaire [22]. Current and past smoking was assessed using questions modified from the Finrisk 2012 survey [23]. Conditioning and everyday physical activity was assessed by questions modified from physical activity questionnaires used in the Finrisk survey [24,25], the Kuopio Ischaemic Heart Disease Risk Factor study [26] and the International Physical Activity Questionnaire [27]. The total duration of conditioning and everyday physical activity was calculated by multiplying the number of sessions of physical activity per week by the duration of each session of physical activity. Total physical activity was calculated by adding conditioning and everyday physical activity together.

#### 2.3.5. Anthropometric and Clinical Measurements

Height, weight, waist circumference and resting blood pressure were measured in healthcare centres by the nurse using standard protocols with calibrated instruments. Body height was measured with the participant standing in the standard anatomical position without shoes in scaled height meters to the nearest 1 cm. Body weight was measured in light indoor clothing by digital scales to the nearest 0.1 kg. Moreover, waist circumference was measured in a standing position on bare skin at the end of normal exhalation and at the mid-distance between the bottom of the rib cage and the top of the iliac crest to the nearest 1 cm. BMI was calculated by dividing the person’s weight in kilograms by the square of his/her height in meters. Additionally, blood pressure was measured twice after a 5 min rest at 2 min intervals from the right arm in a sitting position with standard automatic sphygmomanometers to the nearest 1 mmHg. Two measurements of blood pressure were averaged to obtain the mean of both systolic and diastolic blood pressure.

#### 2.3.6. Biochemical Measurements

Laboratory measurements were taken by laboratory nurses at local healthcare centres after a twelve-hour overnight fast and analysed in local, standardized, quality-controlled clinical laboratories of the three study regions, except plasma insulin, which was analysed in the laboratory of the University of Eastern Finland. Laboratory measurements included a 2 h OGTT after the ingestion of 75 g of glucose (fasting, 30 min, and 2 h plasma glucose and insulin concentrations), glycated haemoglobin (HbA_1c_) as well as fasting concentrations of plasma total, LDL (low-density lipoprotein) and HDL cholesterol and triglycerides.

#### 2.3.7. Assessments of Type 2 Diabetes, Insulin Sensitivity and Secretion and Metabolic Syndrome

Having new, previously undiagnosed, type 2 diabetes (new type 2 diabetes) was defined by the criteria of the American Diabetes Association (ADA, fasting plasma glucose ≥7.0 mmol/L or 2 h plasma glucose ≥11.1 mmol/L) [28] based on one 2 h OGTT. Moreover, isolated IFG (iIFG, fasting plasma glucose 5.6–6.9 mmol/L); isolated IGT (iIGT, 2 h plasma glucose 7.8–11.0 mmol/L) and combined IFG/IGT were determined [28]. The Matsuda insulin sensitivity index (Matsuda ISI) and the disposition index were calculated based on glucose and insulin concentrations at 0, 30 and 120 min according to widely used formulas [29,30]. Matsuda ISI was used as a surrogate measure of peripheral insulin sensitivity and disposition index as a measure of early-phase insulin secretion, as previously validated against the 2 h OGTT [30,31]. Additionally, the participants were defined as having metabolic syndrome if they had at least three of five risk factors of the Joint Interim Statement criteria [32]. These were (1) elevated waist circumference (≥88 cm in women, ≥102 cm in men), (2) elevated plasma triglyceride concentration (≥1.7 mmol/L), (3) reduced plasma HDL cholesterol concentration (<1.0 mmol/L in men, <1.3 mmol/L in women); (4) elevated systolic or diastolic blood pressure (≥130/85 mmHg) or antihypertensive drug treatment and (5) elevated fasting plasma glucose concentration (≥5.6 mmol/L) or antiglycaemic drug treatment [32]. The use of drug treatment for reduced plasma HDL cholesterol concentration or elevated plasma triglyceride concentration was not included in the assessment.

### 2.4. Statistical Analyses

Statistical analyses were performed with the SPSS Statistics, Version 25.0 (IBM, Armonk, NY, USA). Continuous variables are presented as means (±SD, standard deviation), and categorical variables as frequencies (%). Two-sided *p* values < 0.05 were considered statistically significant. Non-normally distributed metabolic variables were ln-logarithm transformed. Differences between ECPs and NECPs were analysed for continuous normally distributed variables by Student’s *t* test (Independent samples *t* test), for continuous variables with skewed distributions by the Mann–Whitney *U* test and for categorical variables by the χ^2^. The differences in metabolic factors between the ECPs and NECPs were also analysed by ANCOVA adjusted for potential confounding factors, age and sex, as covariates.

We used logistic regression models to calculate odds ratios (ORs, 95% CI, confidence interval) for new type 2 diabetes; BMI ≥30 kg/m^2^; abdominal obesity (waist circumference for women ≥88 cm and for men ≥ 102 cm); abnormal glucose tolerance (fasting glucose ≥5.6 mmol/L and 2 h OGTT ≥8 mmol/L); iIFG; iIGT; combined IFG/IGT; elevated total cholesterol (>5mmol/L); elevated LDL cholesterol (>3 mmol/L); reduced HDL cholesterol (women <1.2 mmol/L; men <1.0 mmol/L); hypertriglyceridaemia (>1.7 mmol/L); elevated blood pressure (≥140/90 mmHg) and metabolic syndrome among ECPs compared with NECPs. Participants with normal glucose tolerance were used as a reference group for calculating ORs for new type 2 diabetes, abnormal glucose tolerance, iIFG, iIGT and IFG/IGT. The data were adjusted for age and sex, and additionally for a native country (Finland or other), study region (Northern Savo, Southern Carelia or Päijät-Häme), marital status (married/cohabiting/in a registered relationship or divorced/widowed/not married), family history of type 2 diabetes (yes or no), annual household gross income (€0–44,999/year or ≥ €45,000/year) and the use of lipid-lowering drugs (yes or no). Odds for elevated blood pressure were also adjusted for the use of antihypertensive drugs (yes or no). We used a linear regression model for more detailed analysis of the associations of eating competence, and especially its subcomponents, with the metabolic risk factors of type 2 diabetes by simultaneously entering all subcomponents into the regression models adjusted for age and sex.

## 3. Results

### 3.1. Eating Competence, Sociodemographics and Lifestyle Patterns of Study Participants

**Figure 1:**
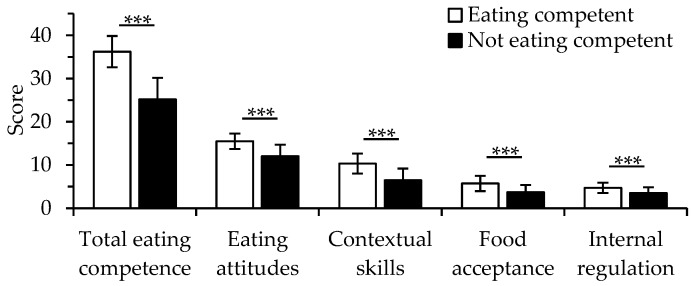
The means (±SD, standard deviation) of total eating competence scores and its subscores in eating competent (*n* = 1159) and not eating competent participants (*n* = 1988). Possible score ranges are 0–48 for total eating competence, 0–18 for eating attitudes, 0–15 for contextual skills, 0–9 for food acceptance and 0–6 for internal regulation. *** *p* value < 0.001.

The total eating competence score ranged between 0 and 48 (mean 29.2, SD 7.0; median 29.0). In total, 37% of the participants were defined as eating competent participants (ECPs, ≥32 points, Table 1). ECPs had higher scores than not eating competent participants (NECPs) in all four subcomponents of eating competence (*p* < 0.001 for all, Figure 1). Characteristics of the ECPs and NECPs are provided in Table 1. ECPs were older (*p* < 0.001), more likely to be born in Finland (*p* = 0.003), had higher household income level (*p* = 0.002), were more educated (*p* = 0.027) and were more likely to use lipid-lowering drugs (*p* = 0.004) compared with NECPs (Table 1). Moreover, compared with NECPs, ECPs were less likely to be smokers (*p* < 0.001) and reported more physical activity (*p* < 0.001), longer sleep duration on weekdays (*p* < 0.001) and better sleep quality (*p* < 0.001) (Table 2). Additionally, ECPs were more likely to consume three main meals (*p* < 0.001), vegetarian dishes (*p* = 0.001), vegetables and fruit (*p* < 0.001) and nuts and seeds (*p* < 0.001), and were less likely to consume sweet desserts and fast food than NECPs (*p* = 0.001) (Table 2). Moreover, ECPs used mostly oil-based fats (*p* = 0.016) and consumed less processed meat products (*p* < 0.001) and sugary beverages (*p* = 0.009) but more alcohol (*p* < 0.001) than NECPs.

### 3.2. Eating Competence and Metabolic Risk Factors

ECPs had a lower BMI (*p* < 0.001, without adjustments) and waist circumference (*p* < 0.001), but higher HbA_1c_ (*p* = 0.024) and systolic blood pressure (*p* = 0.001) compared with NECPs (Table 3). While there were no differences in fasting, 30 min or 2 h plasma glucose between ECPs and NECPs, ECPs had lower fasting (*p* < 0.001) and 30 min plasma insulin (*p* = 0.024) than NECPs. Furthermore, ECPs had a higher Matsuda ISI than NECPs (*p* < 0.001), but no difference was found in the disposition index between ECPs and NECPs. Moreover, ECPs had higher plasma HDL cholesterol (*p* < 0.001) and lower plasma triglyceride (*p* < 0.008) compared with NECPs. After adjustments for age and sex, all these differences remained statistically significant, except the differences in HbA_1c_ and systolic blood pressure (Table 3). Moreover, ECPs had lower 2 h plasma glucose than NECPs after adjustment for age and sex (*p* = 0.022). When we further adjusted the associations for BMI, the differences in metabolic risk factors between ECPs and NECPs were no longer statistically significant (data not shown).

**Figure 2:**
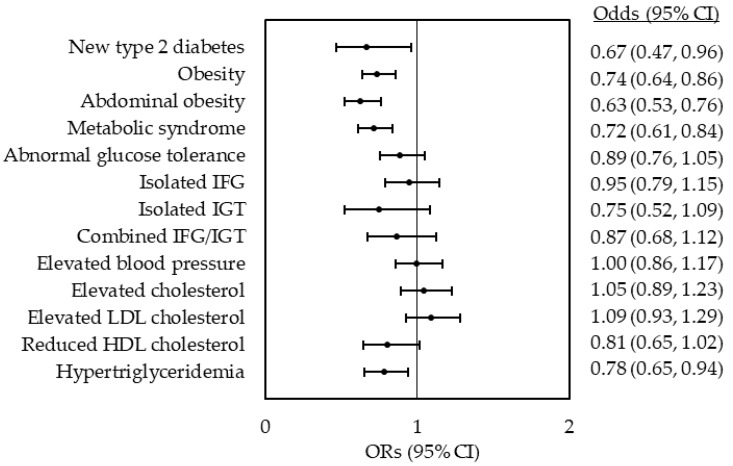
ORs (odds ratios) for the risk of type 2 diabetes and type 2 diabetes risk factors in eating competent participants (ECPs) (score ≥32) compared with not eating competent participants (NECPs) (score <32). Results are presented as point estimates with 95% CIs (confidence intervals) and adjusted for age, sex, study region, native country, marital status, annual household gross income level, family history of type 2 diabetes and use of lipid-lowering drugs. Elevated blood pressure was further adjusted for the use of antihypertensive drugs. Participants with normal glucose tolerance (*n* = 1297) were used as a reference group to analyse the risk for type 2 diabetes (*n* = 176), abnormal glucose tolerance (*n* = 1764), isolated IFG (*n* = 1041) and IGT (*n* = 153) and combined IFG/IGT (*n* = 394).

ECPs were 33% less likely to have new type 2 diabetes (*p* < 0.029), 26% less likely to be obese (*p* < 0.001), 37% less likely to have abdominal obesity (*p* < 0.001), 28% less likely to have a metabolic syndrome (*p* < 0.001) and 21% less likely to have hypertriglyceridaemia (*p* = 0.009) compared with NECPs adjusted for study region, native country, marital status, household gross income, family history of type 2 diabetes and use of lipid-lowering drugs (Figure 2). After further adjustment for BMI, these associations, except for the one with a metabolic syndrome (OR 0.84, 95% CI 0.70, 0.99), were no longer statistically significant (data not shown).

### 3.3. Subcomponents of Eating Competence and Metabolic Risk Factors

Of the four subscores of the total eating competence score, contextual skills score had the strongest associations with metabolic factors (Table 4). The contextual skills score was inversely associated with factors such as BMI (*p* < 0.001), waist circumference (*p* < 0.001), fasting insulin (*p* < 0.001) and triglycerides (*p* < 0.001) and positively associated with the Matsuda ISI (*p* < 0.001) when adjusted for age and sex. The associations of subscores of the total eating competence other than the contextual skills score with metabolic factors were weaker (*p* > 0.001 for all, Table 4).

## 4. Discussion

We investigated the associations of eating competence with reported dietary intake and physical activity, and with metabolic risk factors for type 2 diabetes, in a large group of Finnish adults screened for type 2 diabetes risk. Overall, eating competence, an indicator of positive eating-related behaviour and attitude, was associated with health-promoting lifestyle, a lower likelihood of having new type 2 diabetes, metabolic syndrome and its main components: abdominal obesity, insulin resistance, impaired glucose tolerance, hypertriglyceridaemia and decreased HDL cholesterol. The associations of eating competence with these metabolic abnormalities were at least partly mediated by adiposity because statistically significant associations were not observed after adjustment for BMI, except for the metabolic syndrome. Contextual skills of all four eating competence subcomponents were most strongly associated with metabolic risk factors.

In this study, 37% of participants were classified as eating competent among individuals at risk for type 2 diabetes. In previous studies, the proportion of ECPs has varied between 18% and 53% [6,7,9,10,12,14,33]. The proportion of ECPs in the current study was lower than among Spanish elderly individuals with increased risk for cardiovascular diseases [10], US adults who were mostly females and well educated [6] and among US adults who had hypercholesterolemia [9]. On the other hand, the proportion of ECPs was higher than among stressed and overweight Finnish adults (20%) [33] and overweight and obese well-educated US women (18%) [12]. In this study participants were slightly more educated than Finnish population in general [34] and the mean age was rather high, which has been associated with higher eating competence [6,11], as seen also in our study (Table 1).

We found that ECPs had smaller BMI and waist circumference than NECPs. This is important because increased BMI and waist circumference are the most important risk factors for type 2 diabetes [35,36,37]. Measuring waist circumference particularly indicates abdominal obesity, and waist circumference may be even a better predictor of type 2 diabetes risk than BMI [38]. Our results are well in line with previous publications that have reported an inverse association of eating competence with BMI in most [6,7,12,13,15,39], although not in all studies [9,10]. In a previous one-year weight loss intervention, eating competence increased as BMI decreased among obese individuals [12]. These suggest that enhancing eating competence may contribute to weight loss and subsequently, improve the metabolic profile of an individual.

We observed a lower likelihood of having new type 2 diabetes and a lower 2 h glucose concentration in ECPs than in NECPs. No differences in the risk of other prediabetic states, such as IFG, were observed between the eating competence groups. Earlier, in a study conducted among 638 elderly Spanish individuals with a high risk for cardiovascular diseases, eating competence was associated with a lower risk of IFG [10]. One reason for the different associations of eating competence with prediabetic states other than increased 2 h glucose may result from the fact that all participants in the present study were screened for a high risk of type 2 diabetes, which could have reduced variance in the measures of prediabetic states among the participants. Moreover, a rather small number of participants with IGT (*n* = 153) in our study could reduce the power to observe an association between eating competence and IGT.

In previous studies, components of metabolic syndrome, such as low plasma triglycerides [9] and high plasma HDL cholesterol [10], have been associated with eating competence. This was also seen in the present study (Table 3). The novel result of our study was the association of eating competence with decreased risk of metabolic syndrome, as defined by the Joint Interim Statement criteria [32], and particularly, improved peripheral insulin sensitivity, as indicated by higher Matsuda ISI and lower fasting insulin in ECPs than in NECPs (Table 3). Notably, the decreased risk of metabolic syndrome in ECPs was independent of BMI as the association was significant even after adjustment for BMI. This suggests that the associations of eating competence with metabolic risk factors of type 2 diabetes are not fully explained by the difference in BMI between ECPs and NECPs.

Our results show that eating competence associates with a healthy diet and higher level of physical activity, which have been associated with a lower risk of type 2 diabetes [35,37]. ECPs had healthier dietary patterns and ate main meals more regularly than NECPs in our study. These observations are also in line with the previous findings [6,7,9,10,11,12]. Moreover, ECPs were physically more active and had better sleep quality than NECPs. Presumably, a healthier diet and greater physical activity in ECPs could explain the beneficial associations of eating competence with lower BMI and insulin sensitivity, as suggested previously [40,41]. The higher alcohol consumption in ECPs than in NECPs could be related to higher education level of ECPs [42].

Interestingly, the current study showed that contextual skills, a subcomponent of eating competence, were most strongly associated with metabolic risk factors for type 2 diabetes. Contextual skills comprise practical matters such as planning meals, considering what food is good for oneself and having regular meals [8]. Eating meals regularly has been found to associate with a better quality of diet, whereas a high snacking frequency has been associated with a poorer diet quality [43], and irregular meal frequency with insulin resistance and higher serum total and LDL cholesterol compared with a regular one [44]. Moreover, breakfast skipping has been reported to associate with a higher risk of type 2 diabetes [45], whereas regular eating frequency may have a beneficial effect on body composition [46]. Additionally, there is some preliminary evidence that especially improving contextual skills and positive eating attitudes in weight management interventions are particularly helpful to losing weight [12]. Thus, we suggest that contextual skills were the main determinants behind observed associations between eating competence and metabolic health in our study.

Our study has several strengths. To our knowledge, this is the largest sample so far in a study examining eating competence and its association with metabolic and lifestyle factors related to the risk of type 2 diabetes. In addition, the study includes a comprehensive questionnaire on different lifestyle and health factors, extensive clinical measurements including OGTT and careful adjustments for a multitude of potential risk factors. Moreover, the study was conducted in three different regions in Finland, which reduces selection bias at the population level.

Several limitations should be pointed out as well. First, the causality of the associations cannot be determined with the cross-sectional design of our study. Second, the participants were initially selected based on their increased risk for type 2 diabetes, their average BMI was high, and men were underrepresented, which limits the generalizability of the results to general population. Nonetheless, 63% of women and 72% of men in Finland have BMI > 25 [34], and the study population represents a wide range of at-risk individuals with different backgrounds. The third limitation relates to the method used to examine the dietary intake, which did not allow us to get more detailed information about the composition of a diet related to type 2 diabetes risk, such as intake of dietary fibre or quality of dietary fat. Finally, the diagnosis of type 2 diabetes was assessed based on the results of one OGTT. Therefore, the results concerning the classification of type 2 diabetes should be interpreted with caution.

## 5. Conclusions

The current study shows that eating competence is associated with a better quality of diet, a lower prevalence of new type 2 diabetes, abdominal obesity and metabolic syndrome, as well as better insulin sensitivity. However, the associations of eating competence with new type 2 diabetes or insulin sensitivity were at least partly mediated by BMI.

The findings imply that enhancing eating competence, especially contextual skills for planning and having regular meals, could promote adherence to a healthy diet and thus, in the long term, also support the prevention of type 2 diabetes. Therefore, in the future we need to find ways to enhance eating competence. More intervention and prospective studies are warranted to confirm these suggestions.

## Figures and Tables

**Table 1:** Characteristics of eating competent participants (ECPs, ≥32 points) and not eating competent participants (NECPs, <32 points).

Variable	ECPs (*n* = 1159, 37%)	NECPs (*n* = 1988, 63%)	*p* Value
Age, years (mean ± SD)	57.5 ± 9	53.6 ± 10	<0.001
Sex, female	909 (78)	1587 (80)	0.350
Study region			
Northern Savo	333 (29)	600 (30)	
Southern Karelia	357 (31)	565 (28)	
Päijät-Häme	469 (41)	823 (41)	0.351
Native country (Finland)	1155 (100)	1959 (99)	0.003
Marital status			
Married/cohabiting	879 (76)	1455 (73)	
Unmarried/divorced/widowed	280 (24)	533 (27)	0.101
Annual household gross income			
€0–44,999	451 (39)	885 (45)	
≥€45,000	708 (61)	1103 (56)	0.002
Education level			
Low	88 (7.6)	154 (7.7)	
Medium	287 (24.8)	578 (29.1)	
High	784 (67.6)	1256 (63.2)	0.027
Family history of type 2 diabetes (yes)	917 (79)	1564 (79)	0.767
Use of drugs			
Antihypertensive drugs (yes)	410 (35)	670 (34)	0.340
Lipid-lowering drugs (yes)	228 (20)	312 (16)	0.004
Classifications by plasma glucose ^a^			
Normal glucose tolerance	478 (42)	819 (43)	
Isolated impaired fasting glucose	406 (36)	635 (33)	
Isolated impaired glucose tolerance	51 (5)	102 (5)	
Combined IFG/IGT	146 (13)	248 (13)	
New type 2 diabetes	57 (5)	119 (6)	0.394
FINDRISC ^b^ (mean ± SD)	15.8 ± 4	15.9 ± 4	0.853

Values are reported as *n* (%) for all variables except means (±SD, standard deviation) for age and FINDRISC variables. FINDRISC, the Finnish Diabetes Risk Score; IFG, Impaired fasting glucose; IGT, impaired glucose tolerance. ^a^ *n* = 1138 for ECPs, and *n* = 1923 for NECPs. ^b^ Possible scores range from 0 to 26; 12–14 points indicate a moderate risk, 15–20 points a high risk, and >20 points a very high risk of developing type 2 diabetes within 10 years, *n* = 1155 for ECPs, and *n* = 1978 for NECPs.

**Table 2:** Lifestyle patterns in eating competent participants (ECPs) (≥32 points) and not eating competent participants (NECPs) (<32 points).

Variable	ECPs (*n* = 1159, 37%)	NECPs (*n* = 1988, 63%)	*p* Value
Current smoker, *n* (%)	63 (5)	182 (9)	<0.001
Physical activity, h/week	10.7 ± 10.6 ^a^	8.8 ± 9.1 ^b^	<0.001
Sleep on weekdays, h/day	8.0 ± 1.0 ^a^	7.8 ± 1.1 ^b^	<0.001
Sleep quality (score range 0–10)	8.8 ± 1.7 ^a^	8.5 ± 1.9 ^b^	<0.001
Having main meals every weekday ^c^, *n* (%)	546 (47)	569 (29)	<0.001
Vegetarian dishes/week	1.9 ± 2.1	1.6 ± 1.9	0.003
Eating fast food <1 portion/week, *n* (%)	946 (82)	1379 (69)	<0.001
Vegetables, fruit and berries ≥4 portions/day, *n* (%)	312 (27)	362 (18)	<0.001
Using mostly oil-based fats ^d^, *n* (%)	294 (25)	430 (22)	0.016
Nuts and seeds ≥4 portions (á 30 g)/week, *n* (%)	427 (37)	499 (25)	<0.001
Sweet pastry/pudding/ice cream <4 portions/week, *n* (%)	715 (62)	1088 (55)	<0.001
Processed meat products, g/day	44.6 ± 44 ^a^	54.3 ± 61 ^b^	<0.001
Coffee consumption, dL/day	2.9 ± 2.2 ^a^	2.8 ± 2.3 ^b^	0.130
Sugary beverages, dL/day	0.38 ± 1.2	0.45 ± 1.2 ^b^	0.009
Alcohol consumption, g/day	7.5 ± 12 ^a^	6.4 ± 11 ^b^	<0.001

Values are reported as *n* (%) for categorical variables and means ± SD (standard deviation) for continuous variables. ^a^ Physical activity *n* = 1150; sleep on weekdays *n* = 1155; sleep quality *n* = 1158; processed meat products *n* = 1158; coffee consumption *n* = 1155; alcohol consumption *n* = 1158. ^b^ Physical activity *n* = 1970; sleep on weekdays *n* = 1977; sleep quality *n* = 1983; processed meat products *n* = 1987; sugary beverages *n* = 1987; coffee consumption *n* = 1987; alcohol consumption *n* = 1984. ^c^ Having breakfast, lunch and dinner every weekday. ^d^ Using mostly oil-based cooking fats, salad dressings and margarine as a table spread.

**Table 3:** Metabolic risk factors for type 2 diabetes in eating competent participants (ECPs) (≥32 points) and not eating competent participants (NECPs) (<32 points).

Variable	ECPs (*n* = 1130–1159, 37%)	NECPs (*n* = 1905–1987, 63%)	*p* Value ^a^	*p* Value ^b^
Anthropometric measurements				
BMI, kg/m^2^	30.3 ± 5.3	31.8 ± 5.6	<0.001	<0.001
Waist circumference, cm	100.2 ± 13	103.3 ± 14	<0.001	<0.001
Glucose and insulin metabolism				
Fasting glucose, mmol/L	5.7 ± 0.7	5.7 ± 0.8	0.657	0.376
30 min glucose, OGTT, mmol/L	8.9 ± 1.7	8.8 ± 1.8	0.177	0.914
2 h glucose, OGTT, mmol/L	6.6 ± 2.2	6.7 ± 2.4	0.112	0.022
HbA_1c_, %	5.5 ± 0.4	5.5 ± 0.4	0.024	0.627
HbA_1c_, mmol/mol	36.7 ± 4.4	36.4 ± 4.9	0.017	0.671
Fasting insulin, pmol/L	81.6 ± 52	94.3 ± 82	<0.001	<0.001
30 min insulin, OGTT, pmol/L	511.7 ± 356	555.3 ± 370	0.002	0.024
2 h insulin, OGTT pmol/L	590.7 ± 596	612.8 ± 613	0.451	0.323
Matsuda insulin sensitivity index	12.9 ± 8	12.1 ± 8	<0.001	0.002
Disposition index	420.6 ± 211	424.9 ± 213	0.820	0.387
Lipid metabolism and blood pressure				
Total cholesterol, mmol/L	5.27 ± 1.0	5.22 ± 1.0	0.335	0.983
LDL cholesterol, mmol/L	3.24 ± 0.9	3.25 ± 0.8	0.436	0.346
HDL cholesterol, mmol/L	1.56 ± 0.4	1.50 ± 0.4	<0.001	0.023
Triglycerides, mmol/L	1.37 ± 0.7	1.45 ± 0.8	0.008	0.002
Diastolic blood pressure, mmHg	88.1 ± 10	88.3 ± 10	0.641	0.449
Systolic blood pressure, mmHg	141.3 ± 18	139.0 ± 18	0.001	0.510

Values are means (±SD, standard deviation) and nl-logarithmic transformed for pairwise comparison. BMI, body mass index; OGTT, oral glucose tolerance test; HbA_1c_, glycated haemoglobin; LDL, low-density lipoprotein; HDL, high-density lipoprotein. ^a^ *p* values for the differences in variables between the ECPs and NECPs were obtained by Student *t* test (Independent-samples *t* test). ^b^ *p* values for the differences in variables between the ECPs and NECPs were obtained by ANCOVA for age and sex as covariates.

**Table 4:** Associations of eating competence subcomponents: food attitudes, contextual skills, food acceptance and internal regulation with type 2 diabetes metabolic risk factors.

Metabolic Risk Factor	*n*	Food Attitudes	Contextual Skills	Food Acceptance	Internal Regulation
Anthropometric measurements					
BMI, kg/m^2^	3145	−0.042	−0.150 ***	0.026	0.014
Waist circumference, cm	3146	−0.031	−0.153 ***	0.008	0.029
Glucose and insulin metabolism					
Fasting glucose, mmol/L	3061	0.016	−0.036	0.018	0.006
30 min glucose, OGTT, mmol/L	3056	0.016	−0.030	0.002	−0.001
2 h glucose, OGTT, mmol/L	3061	0.022	−0.028	−0.025	−0.035
HbA_1c_, %	3035	0.048 *	−0.060 **	−0.031	0.010
HbA_1c_, mmol/mol	3035	0.048 *	−0.060 **	−0.031	0.011
Fasting insulin, pmol/L	3057	0.025	−0.131 ***	<0.001	0.018
30 min insulin, OGTT, pmol/L	3055	<0.001	−0.084 ***	−0.010	0.016
2 h insulin, OGTT, pmol/L	3051	0.037	−0.065 **	−0.002	−0.013
Matsuda insulin sensitivity index	3034	−0.025	0.121 ***	0.003	0.027
Disposition index	3034	−0.031	0.049 *	−0.013	0.015
Lipid metabolism and blood pressure					
Total cholesterol, mmol/L	3036	−0.013	−0.046 *	0.052 **	0.056 **
LDL cholesterol, mmol/L	3037	−0.014	−0.052 *	0.046 *	0.057 **
HDL cholesterol, mmol/L	3037	−0.011	0.059 **	0.021	−0.013
Triglycerides, mmol/L	3035	−0.004	−0.088 ***	−0.016	0.035
Diastolic blood pressure, mmHg	3144	0.014	−0.053 *	0.011	0.007
Systolic blood pressure, mmHg	3144	0.027	−0.044 *	0.023	0.019

Values are standardized coefficients beta (β) and *p*-values from linear regression models adjusted for age and sex. BMI, body mass index; OGTT, oral glucose tolerance test; HbA_1c_, glycated haemoglobin; LDL, low-density lipoprotein; HDL, high-density lipoprotein. * *p* value < 0.05, ** *p* value < 0.01, *** *p* value < 0.001.

## Supplementary Materials

The following are available online at https://www.mdpi.com/2072-6643/12/1/104/s1, Supplementary Material: Type 2 diabetes risk assessment form.
