# Supplementary material for: Eating Competence Is Associated with Lower Prevalence of Obesity and Better Insulin Sensitivity in Finnish Adults with Increased Risk for Type 2 Diabetes: The StopDia Study
Source: Nutrients. 2019 Dec 30;12(1):104. doi: 10.3390/nu12010104 (PMC7019577; doi:10.3390/nu12010104)
Supplement: Supplementary file 1 [file nutrients-12-00104-s001.pdf]

Finnish Diabetes Association

Type 2 diabetes risk assessment form

1. Age

- ☐ Under 45 years (0 p.)
- ☐ 45–54 years (2 p.)
- ☐ 55–64 years (3 p.)
- ☐ Over 64 years (4 p.)

2. Body-mass index

(See reverse of form)

- ☐ Lower than 25 kg/m² (0 p.)
- ☐ 25–30 kg/m² (1 p.)
- ☐ Higher than 30 kg/m² (3 p.)

3. Waist circumference measured below the ribs (usually at the level of the navel)

MEN	WOMEN	
☐ Less than 94 cm	☐ Less than 80 cm	(0 p.)
☐ 94–102 cm	☐ 80–88 cm	(3 p.)
☐ More than 102 cm	☐ More than 88 cm	(4 p.)

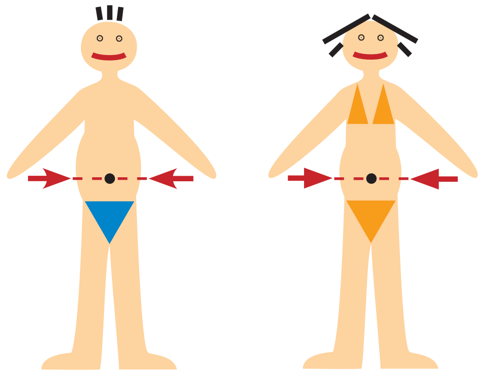

4. Do you usually have daily at least 30 minutes of physical activity at work and/or during leisure time (including normal daily activity)?

- ☐ Yes (0 p.)
- ☐ No (2 p.)

5. How often do you eat vegetables, fruit or berries?

- ☐ Every day (0 p.)
- ☐ Not every day (1 p.)

6. Have you ever taken medication for high blood pressure on regular basis?

- ☐ No (0 p.)
- ☐ Yes (2 p.)

7. Have you ever been found to have high blood glucose (eg in a health examination, during an illness, during pregnancy)?

- ☐ No (0 p.)
- ☐ Yes (5 p.)

8. Have any of the members of your immediate family or other relatives been diagnosed with diabetes (type 1 or type 2)?

- ☐ No (0 p.)
- ☐ Yes: grandparent, aunt, uncle or first cousin (but no own parent, brother, sister or child) (3 p.)
- ☐ Yes: parent, brother, sister or own child (5 p.)

Total Risk Score

☐ **The risk of developing type 2 diabetes within 10 years is**

**Lower than 7**	**Low:** estimated 1 in 100 will develope disease
**7–11**	**Slightly elevated:** estimated 1 in 25 will develope disease
**12–14**	**Moderate:** estimated 1 in 6 will develope disease
**15–20**	**High:** estimated 1 in 3 will develope disease
**Higher than 20**	**Very high:** estimated 1 in 2 will develope disease

WHAT CAN YOU DO TO LOWER YOUR RISK OF DEVELOPING TYPE 2 DIABETES?

You can't do anything about your age or your genetic predisposition. On the other hand, the rest of the factors predisposing to diabetes, such as overweightness, abdominal obesity, sedentary lifestyle, eating habits and smoking, are up to you. Your lifestyle choices can completely prevent type 2 diabetes or at least delay its onset until a much greater age.

If there is diabetes in your family, you should be careful not to put on weight over the years. Growth of the waistline, in particular, increases the risk of diabetes, whereas regular moderate physical activity will lower the risk. You should also pay attention to your diet: take care to eat plenty of fibre-rich cereal products and vegetables every day. Omit excess hard fats from your diet and favour soft vegetable fats.

Early stages of type 2 diabetes seldom cause any symptoms.

IF YOU SCORED 7–14 POINTS IN THE RISK TEST

● You would be well advised to seriously consider your physical activity and eating habits and pay attention to your weigth, to prevent yourself from developing diabetes.

● Please contact a public-health nurse or your own doctor for further guidance and tests.

IF YOU SCORED 15–20 POINTS IN THE RISK TEST

● You should have your blood glucose measured (both fasting value and value after a dose of glucose or a meal) to determine if you have diabetes without symptoms.

IF YOU SCORED OVER 20 POINTS IN THE RISK TEST

● Please contact a public-health nurse or your own doctor soon.

The test is designed by Professor Jaakko Tuomilehto, Department of Public Health, University of Helsinki, and Dr Jaana Lindström, National Institute for Health and Welfare, Finland, and it is published by the Finnish Diabetes Association.

BMI

The body-mass index is used to assess whether a person is normal weight or not. The index is calculated by dividing body weight (kg) by the square of body height (m). For example, if your height is 165 cm and your weight 70 kg, your BMI will be 25.7 [70/(1.65 x 1.65)=25.7].

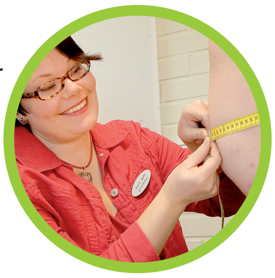

If your BMI is 25–30, you will benefit from losing weight; at least you should take care that your weight doesn't increase beyond this. If your BMI is higher than 30, the adverse health effects of obesity will start to show, and it will be essential to lose weight.

BMI-chart

Height (cm)

200	13	13	14	14	15	15	16	16	17	17	18	18	19	19	20	20	21	21	22	22	23	23	24	24	25	25	26	26	27	27	28	28	29	29	30	30	31	31	32	32	33	33	34	34
198	13	13	14	14	15	15	16	16	17	17	18	18	19	19	20	20	21	21	22	22	23	23	24	24	25	26	26	26	27	28	28	29	29	30	30	31	31	32	32	33	33	34	34	35
196	13	14	14	15	15	16	16	17	17	18	18	19	19	20	20	21	21	22	22	23	23	24	24	25	26	26	27	27	28	28	29	29	30	30	31	31	32	32	33	33	34	34	35	35
194	13	14	14	15	15	15	16	17	18	18	19	19	19	20	21	21	22	22	22	23	23	24	25	26	26	27	27	28	28	29	29	30	30	31	31	32	32	33	34	34	35	35	36	36
192	14	14	15	15	16	16	17	17	18	18	19	20	20	21	21	22	22	23	23	24	24	25	25	26	27	27	28	28	29	29	30	30	31	32	32	33	33	34	34	35	35	36	36	37
190	14	14	15	16	16	17	17	18	18	19	19	20	20	21	22	22	23	23	24	24	25	25	26	27	27	28	28	28	29	30	30	31	32	32	33	33	34	34	35	36	36	37	37	38
188	14	15	15	16	16	17	18	18	19	19	20	20	21	22	22	23	23	24	24	25	25	26	27	27	28	28	29	29	30	31	32	32	32	33	33	34	35	35	36	36	37	37	38	39
186	14	15	16	16	17	17	18	18	19	20	20	21	21	22	23	23	24	24	25	25	26	27	27	28	28	29	29	30	31	31	32	32	33	34	34	35	35	36	36	37	38	38	39	39
184	15	15	16	17	17	18	18	19	19	20	21	21	22	22	23	24	24	25	25	26	27	27	28	28	29	29	30	31	31	32	32	33	34	34	35	35	36	37	37	38	38	39	40	40
182	15	16	16	17	18	18	19	19	20	21	21	22	22	23	24	24	25	25	26	27	27	28	28	29	30	30	31	31	32	33	33	34	34	35	36	36	37	37	38	39	39	40	41	41
180	15	16	17	17	18	19	19	20	20	21	22	22	23	23	24	25	25	26	27	27	28	28	29	30	30	31	31	32	33	33	34	35	35	36	36	37	38	38	39	40	40	41	41	42
178	16	16	17	18	18	19	20	20	21	21	22	23	23	24	25	25	26	27	27	28	28	29	30	30	31	32	32	33	33	34	35	35	36	37	37	38	39	39	40	40	41	42	42	43
176	16	17	17	18	19	19	20	21	21	22	23	23	24	25	25	26	26	27	28	28	29	30	30	31	32	32	33	34	34	35	36	36	37	37	38	39	39	40	41	41	42	43	43	44
174	17	17	18	18	19	20	20	21	22	22	23	24	24	25	26	26	27	28	28	29	30	30	31	32	32	33	34	34	35	36	36	37	38	38	39	40	40	41	42	42	43	44	44	45
172	17	18	18	19	20	20	21	22	22	23	24	24	25	26	26	27	28	28	29	30	30	31	32	32	33	34	34	35	36	37	37	38	39	39	40	41	41	42	43	43	44	45	45	46
170	17	18	19	19	20	21	21	22	23	24	24	25	26	26	27	28	28	29	30	30	31	32	33	33	34	35	35	36	37	37	38	39	39	40	41	42	42	43	44	44	45	46	46	47
168	18	18	19	20	21	21	22	23	23	24	25	26	26	27	28	28	29	30	30	31	32	33	33	34	35	35	36	37	38	38	39	40	40	41	42	43	43	44	45	45	46	47	48	48
166	18	19	20	20	21	22	22	23	24	25	25	26	27	28	28	29	30	30	31	32	33	33	34	35	36	36	37	38	38	39	40	41	41	42	43	44	44	45	46	47	47	48	49	49
164	19	19	20	21	22	22	23	24	25	25	26	27	28	28	29	30	30	31	32	33	33	34	35	36	36	37	38	39	39	40	41	42	42	43	44	45	45	46	47	48	48	49	50	51
162	19	20	21	21	22	23	24	24	25	26	27	27	28	29	30	30	31	32	33	34	34	35	36	37	37	38	39	40	40	41	42	43	43	44	45	46	47	47	48	49	50	50	51	52
160	20	20	21	22	23	23	24	25	26	27	27	28	29	30	30	31	32	33	34	34	35	36	37	38	38	39	40	41	41	42	43	44	45	45	46	47	48	48	49	50	51	52	52	53
158	20	21	22	22	23	24	25	26	26	27	28	29	30	30	31	32	33	34	34	35	36	37	38	38	39	40	41	42	42	43	44	45	46	47	47	48	49	50	51	51	52	53	54	55
156	21	21	22	23	24	25	25	26	27	28	29	30	30	31	32	33	34	35	35	36	37	38	39	39	40	41	42	43	44	44	45	46	47	48	49	49	50	51	52	53	53	54	55	56
154	21	22	23	24	24	25	26	27	28	29	30	30	31	32	33	34	35	35	36	37	38	39	40	40	41	42	43	44	45	46	46	47	48	49	50	51	51	52	53	54	55	56	57	57
152	22	23	23	24	25	26	27	28	29	29	30	31	32	33	34	35	35	36	37	38	39	40	41	42	42	43	44	45	46	47	48	49	49	50	51	52	53	54	55	55	56	57	58	59
	normal weight						mild obesity						marked obesity						severe obesity															morbid obesity										
	50	52	54	56	58	60	62	64	66	68	70	72	74	76	78	80	82	84	86	88	90	92	94	96	98	100	102	104	106	108	110	112	114	116	118	120	122	124	126	128	130	132	134	136

Weight (kg)

1/2010
